# Determinants of glycaemic control among type 2 diabetes mellitus patients in Northern State of Kedah, Malaysia: a cross-sectional analysis of 5 years national diabetes registry 2014-2018

**DOI:** 10.11604/pamj.2021.39.206.30410

**Published:** 2021-07-21

**Authors:** Mohd Rohaizat Hassan, Mohd Nazrin Jamhari, Firdaus Hayati, Norfazilah Ahmad, Mohd'Ammar Ihsan Ahmad Zamzuri, Azmawati Mohammed Nawi, Khaironi Yatim Sharif, Muhammad Sufri, Shahrul Bariyah Ahmad, Norhizan Ismail, Syed Sharizman Syed Abdul Rahim, Mohammad Saffree Jeffree

**Affiliations:** 1 Department of Community Health, Faculty of Medicine, Universiti Kebangsaan Malaysia, Jalan Yaacob Latiff, Bandar Tun Razak, Kuala Lumpur, Malaysia,; 2 Department of Surgery, Faculty of Medicine and Health Sciences, Universiti Malaysia Sabah, Kota Kinabalu, Sabah, Malaysia,; 3 Department of Software Engineering and Information System, Faculty of Computer Science and Information Technology, Universiti Putra Malaysia, Serdang, Selangor Darul Ehsan, Malaysia,; 4 Kedah State Health Department, Ministry of Health Malaysia, Jalan Kuala Kedah, Simpang Kuala, Alor Setar, Kedah, Malaysia,; 5 Department of Community and Family Medicine, Faculty of Medicine and Health Sciences, Universiti Malaysia Sabah, Kota Kinabalu, Sabah, Malaysia

**Keywords:** Diabetes, glycaemic control, obesity, type 2 DM, waist circumference

## Abstract

**Introduction:**

type 2 diabetes mellitus has become a global public health crisis. The increment in the cases has contributed significantly to the parallel increase in the prevalence of overweight and obesity. This paper aimed to analyse the relationship between lipid profile, waist circumference and body mass index (BMI) with the glycaemic control of the diabetes patients in Kedah.

**Methods:**

a cross-sectional study was conducted, using the Kedah audit samples data extracted from the National Diabetes Registry (NDR) from the year 2014 to 2018. A total of 25,062 registered type 2 diabetes mellitus patients were selected using the inclusion and exclusion criteria from the registry. Only patients with complete data on their HbA1C, lipid profile, waist circumference and BMI were analysed using SPSS version 21.

**Results:**

the means for the age, BMI and waist circumference of the samples were 61.5 (±10.85) years, 27.3 (±5.05) kg/m^2^ and 89.46 (±13.58) cm, respectively. Poor glycaemic control (HbA1c>6.5%) was observed in 72.7% of the patients, with females having poorer glycaemic control. The BMI and waist circumference were found to be significantly associated with glycaemic control (P<0.001). The total cholesterol, triglycerides and low-density lipoproteins values showed positive correlation with glycaemic control (r = 0.178, 0.157, 0.145, p<0.001), while high-density lipoproteins values are negatively correlated (r = -0.019, p<0.001).

**Conclusion:**

implementing lifestyle changes such as physical activity and dietary modifications are important in the management of BMI, waist circumference and body lipids, which in turn results in improved glycaemic control.

## Introduction

Diabetes mellitus is characterised by chronic hyperglycaemia and is closely associated with serious health issues, including neuropathy, nephropathy, retinopathy, and obesity [1]. The global prevalence of type 2 diabetes mellitus patients has increased from 8.1% to 9.6% in Asians and from 6.0% to 7.9% in Caucasians [2]. Type 2 diabetes mellitus also has become a global public health crisis that threatens the economies of all nations, particularly the developing countries [3]. Fuelled by rapid urbanisation, nutrition transition, and increasingly sedentary lifestyles, the epidemic has grown in parallel with the worldwide rise in obesity [4]. The fifth Malaysian National Health Morbidity Survey (NHMS) 2015 reported the prevalence of patients with type 2 diabetes mellitus had increased to 17.5% in Malaysia as compared with the fourth NHMS, which reported a prevalence of 15.2% in 2011 [5]. Kedah has become the state with the highest prevalence of diabetes mellitus (25.4%) and hypertension (37.5%) in Malaysia, the second-highest prevalence of hypercholesterolemia (53.5%) and fifth-highest prevalence of obesity (33.2%) [5].

The increase in the prevalence of type 2 diabetes mellitus patients has contributed significantly to the parallel increase in the prevalence of overweight and obesity. The overall prevalence of abdominal obesity in Malaysia, measured by waist circumference, has been reported between 55.6% and 57.4% [6]. Epidemiologic studies investigating abdominal obesity in Malaysia have consistently shown an ethnic trend similar to that seen in patients with type 2 diabetes mellitus with prevalence being highest among Asian Indians (65.5%-68.8%), followed by Malays (55.1%-60.6%), Chinese (49.5%-51.1%), and other indigenous groups (44.9%-48.3%) [7]. Obesity and diabetes have become inseparable where there has been a growing prevalence of abdominal obesity in people with type 2 diabetes mellitus; indeed, obesity is observed in 75% of Malaysians with type 2 diabetes mellitus. Additionally, in the 2008 Malaysia diabcare study, an undesirable waist circumference was reported in a higher proportion among women (80cm in 89.4%) than men (90cm in 73.7%) with type 2 diabetes mellitus. This study also found that 72% of people with type 2 diabetes mellitus were also obese with a mean body mass index (BMI) of 27.8kg/m^2^ [8].

Glycaemic control is known to be influenced by a number of factors, which can be categorised into several groups including patient-related factors (e.g. ethnicity, age, gender and non-adherence to medication), disease-related factors (e.g. longer duration of diabetes and the presence of metabolic syndrome), treatment-related factors (e.g. physical inactivity and monotherapy with oral hypoglycaemic agents), healthcare provider-related factors (e.g. reluctance to start exogenous insulin therapy), health facility-related factors (e.g. lack of certain types of medications or staff shortage) and socioeconomic factors (e.g. employment status, income and level of education) [9-11]. Obesity is associated with an increased risk of developing insulin resistance and type 2 diabetes mellitus. In obese individuals, adipose tissue releases increased amounts of non-esterified fatty acids, glycerol, hormones, pro-inflammatory cytokines and other factors that are involved in the development of insulin resistance. When insulin resistance is accompanied by dysfunction of pancreatic islet β-cells (the cells that release insulin), it can cause failure to control blood glucose levels [12-14]. Since there are still gaps of knowledge on the relationship between lipid compositions, waist circumference, BMI and glycaemic control, we aim to analyse the relationship between waist circumference, BMI and lipid profile with the glycaemic control among patients with type 2 diabetes in Kedah, Malaysia.

## Methods

**Data collection:** the NDR database contains information about patients with diabetes receiving care at the participating Health Clinics. An electronic, web-based data-entry system has been used for this purpose. Data collection is performed by the clinic staff, using Audit Form (NCD/Audit/version_3.0/2008) consisting of 6 main components: (i) patient and facility demographic, date of diagnosis and type of diabetes, (ii) clinical investigation results, (iii) complications, (iv) comorbidities, (v) anti-diabetic drug use, and (vi) drug treatments for concomitant conditions. Ethical issues are the most important component of this study. Only data on screening and management was taken from the registry. For demographic data, only patient´s identification numbers with the name of the health clinics and districts were derived from the registry. No full names were taken to preserve confidentiality.

**Sampling method:** the samples selected for the audit are all patients (controlled and poorly controlled) who were actively followed up (at least once a year) in any public health clinics across Kedah during the particular year and they must be registered under the NDR. According to Malaysian clinical practice guideline: management of type 2 diabetes mellitus (5^th^ Edition), those who have HbA1C ≤6.5% are considered as good controlled and those who are 6.6% and above are considered poorly controlled. For this study, the inclusion criteria of the sample selection are among those with completed data of waist circumference, BMI, lipid profile and HbA1C. However, patients who were transferred out, defaulted follow up or dead were excluded in this study sample. Sample calculations have been automatically performed by the web-based application and the patients were randomly selected from the registry. Different samples of patients are drawn every year. Patients sampled in the previous year have an equal chance of being selected in the subsequent years. The sample size is determined by the number of active patients with type 2 diabetes mellitus within a district. The audit was done in the one month, between 1^st^ August until 30^th^ August every year by reviewing the selected samples or patient´s diabetic book.

**Statistical analysis:** data were analysed by using SPSS (version 21.0, IBM). Categorical data were expressed as frequencies and percentages, while numerical data were expressed as means and standard deviations. Bivariate analysis was conducted (t-test, chi-square and Pearson´s Correlation test) to determine the association between the independent variables on the glycaemic control. All explanatory variables that achieved a p-value <0.25 from bivariate analysis were subsequently included in the multivariable analysis [15]. Logistic regression analysis was performed to obtain the crude odds ratio, adjusted odds ratio and their 95% confidence interval, as well as to determine factors associated with glycaemic control. The significance level in this study was set at p<0.05.

**Ethical consideration:** ethical approval was obtained from the Medical research and ethic committee of Malaysia (NIH.800-4/4/1 Jld. 79(40)). Permission on analyzing NDR data was obtained from State Health Director of Kedah State Health Department.

## Results

**General characteristics:** the total number of NDR Audit samples for the state of Kedah from the year 2014 until 2018 is 80,762 samples. Following the inclusion and exclusion criteria mentioned above, we finalised the 25,062 samples selected to be analysed. The selected samples are the completed data of waist circumference, BMI measurement, lipid profile and HbA1C. From the descriptive analysis, we can see that 64.8% of the samples selected are female and of Malay ethnicity (83.4%). The mean age of the samples is 61.5 (±10.85) years old, while the mean waist circumference of the samples is 89.46 (±13.58) cm. Majority of the samples are overweight (66.4%), followed by normal weight and underweight. In terms of the lipid profile, the mean of total cholesterol is 5.27 (±1.27) mmol/L, triglycerides (TG) 1.87 (±1.08) mmol/L, high-density lipoproteins (HDL) 1.29 (±0.41) mmol/L, low-density lipoproteins (LDL) 3.10 (±1.14) mmol/L.

**Bivariate analysis:** the result of the bivariate analysis was shown in Table 1. Samples with significantly good diabetes control (HbA1c ≤6.5%) are among the males (28.84%), Indian ethnicity (36.81%) and underweight (36.03%) [16]. In terms of age, older patients tend to have poorer glycaemic control (HbA1C >6.5%) as compared to the younger group [17]. Patients with bigger waist circumference significantly had poor glycaemic control compared to those who have smaller waist circumference. Body mass index (BMI) has also been found to be significantly associated with glycaemic control. Underweight patients significantly had good glycaemic control compared to those who are of normal weight and are overweight. Samples with higher total cholesterol, TG and LDL were significantly associated with poor glycaemic control, while those with higher HDL significantly associated with having good glycaemic control.

**Table 1:** association between age, gender, ethnicity, waist circumference and BMI with glycemic control (HbA1C)

Variables	Glycemic control (%)		Statistical value	P value
	Good	Poor		
Age (in years) *	64.18 (±11.0)	60.49 (±10.62)	23.86	<0.001^a^
Gender			16.92	<0.001^b^
Male	28.84	71.16		
Female	26.42	73.58		
Race			108.12	<0.001^b^
Malay	26.64	73.46		
Indian	36.81	63.19		
Chinese	25.06	74.94		
Others	33.84	66.16		
Waist circumference*	88.62 (±12.99)	89.77 (±13.78)	-6.12	<0.001^a^
BMI			109.23	<0.001^b^
Underweight	36.03	63.97		
Normal weight	31.04	68.96		
Overweight	25.23	74.77		
Lipid profile*				<0.001^a^
Total Chol	5.04 (±1.18)	5.36 (±1.30)	-18.65	
Triglyceride	1.67 (±0.94)	1.94 (±1.12)	-19.33	
HDL	1.31 (±0.43)	1.28 (±0.40)	4.22	
LDL	2.99 (±1.09)	3.21 (±1.16)	-13.77	

a t-test,^b^ Chi-square *mean (±sd), BMI: body mass index, HDL: high density lipoprotein, LDL: low density lipoprotein

**Determinants of glycemic control among patients with type 2 diabetes:**Table 2 shows a significant correlation between all four types of lipids with glycaemic control (HbA1C). Patients who have high TG, LDL and total cholesterol have a significantly higher risk to have poor glycaemic control (positive r-value, p<0.001), while patients with high HDL were significantly found to have good diabetes control (r = -0.019, p<0.001). In a multivariate analysis (Table 3), age [aOR = 0.972, (95% CI 0.969,0.974)], male gender [aOR = 0.930 (95% CI 0.8760.988)], and HDL [aOR = 0.898(95% CI 0.8280.973)] were found to be the predicting factors for good glycaemic control among patients with type 2 diabetes in this analysis. Meanwhile, the BMI, total cholesterol and LDL were found to be not significant.

**Table 2:** correlation between lipid profile with glycemic control (HbA1C)

Variable	Mean (SD)	r	r^2^	P value
TG	1.87(1.087)	0.157	0.025	<0.001
HDL	1.29(0.409)	-0.019	0.0004	<0.001
LDL	3.15(1.142)	0.145	0.021	<0.001
Total cholesterol	5.27(1.274)	0.178	0.032	<0.001

TG: triglyceride, HDL: high density lipoprotein, LDL: low density lipoprotein

**Table 3:** factors associated with glycemic control among type 2 diabetes mellitus patients in Kedah

Variables	cOR	95% CI	p value	aOR	95% CI	p value
Age	0.968	0.965-0.971	<0.001	0.972	0.969-0.974	<0.001
Gender						
Female	1					
Male	0.886	0.836-0.979	<0.001	0.930	0.876-0.988	<0.001
BMI						
Underweight	1					
Normal weight	1.251	1.022-1.531	0.03	1.041	0.845-1.283	0.707
Over weight	1.669	1.368-2.038	<0.001	1.179	0.955-1.456	0.126
T. Chol.	1.234	1.206-1.263	<0.001	1.136	1.076-1.199	0.113
TG	1.347	1.304-1.393	<0.001	1.228	1.181-1.278	<0.001
HDL	0.863	0.807-0.922	<0.001	0.898	0.828-0.973	<0.001
LDL	1.190	1.160-1.220	<0.001	1.033	0.978-1.091	0.240

cOR = crude odds ratio, aOR = adjusted odds ratio, BMI: body mass index, T.Chol: total cholesterol, HDL: high density lipoprotein, LDL: low density lipoprotein

## Discussion

Glycaemic control is a critical issue in the clinical management of diabetes and its complications. It has been suggested that intensive glycaemic control is associated with a lower prevalence of microvascular and neuropathic events in patients with type 2 diabetes mellitus [18]. As a chronic life-long debilitating disease, numerous factors including obesity and lipid profile influence glycaemic control. The risk of developing complications can be significantly reduced by optimal glycaemic control. This requires much effort, discipline, skill and knowledge from both sides, the patients and the medical practitioners. Thus, the establishment of the NDR is seen as an excellent initiative from the Ministry of Health (MOH) to monitor the quality of medical care delivered to diabetes patients at primary healthcare facilities and in determining the optimum glycaemic control among diabetes patients nationwide. The methodology used is very relevant and can be representable for the entire population in Malaysia [19]. This study showed the determinants of glycaemic control among type 2 diabetes mellitus patients in Kedah. The association of anthropometry measurement (in this study are waist circumference and BMI) was significant with the glycaemic control (p<0.001). The smaller the waist circumference and the lower the BMI, the patients will have better glycaemic control. This means that it is very important to monitor the waist circumference and BMI measurement of diabetes patients to get the optimum glycaemic control. In terms of age, the study showed that older patients will significantly tend to be poorer at diabetes control compared to younger patients. This may be due to the declining cognitive function of the patients. The presence of cognitive impairment can make it challenging for the clinicians to help their patients to reach individualised glycaemic, blood pressure, and lipid targets. Cognitive dysfunction makes it difficult for patients to perform complex self-care tasks, such as glucose monitoring and adjusting insulin doses [20].

Lipid profiles of diabetes patients also play an important role in determining glycaemic control. Studies strongly show that managing obesity will delay diabetes progression from pre-diabetes to type 2 diabetes mellitus. In type 2 diabetes mellitus patients (with underlying of being overweight or obese), their glycaemic control showed an improvement with modest and sustained weight loss and reduced the need for diabetes medications [21]. It is proved in the study that total cholesterol, HDL, LDL and TG level have significant correlations with glycaemic control (p<0.001). Poor glycaemic control and hypertriglyceridemia are significant biochemical abnormalities in patients with type 2 diabetes mellitus [22]. Our findings were in line with a previous study suggesting that the level of total cholesterol is usually normal or near-normal if glycaemic control is adequate while worsening of this control raises the level. Ozder *et al*. had mentioned in his study that abnormal glucose reading is the commonest metabolic abnormality in people with type 2 diabetes mellitus accompanied by lower HDL levels, elevated LDL, hypercholesterolemia, and hypertriglyceridemia [23]. The World Health Organization (WHO) and International Diabetes Federation (IDF) also described the term of diabetic dyslipidaemia which comprises a triad of raised triglycerides, reduced HDL and excess of small, dense LDL particles. Hussein et al found that the BMI and lipid parameters have been proved to be the significant predictors for metabolic disturbances including diabetes, dyslipidaemia, hypertension, hyperinsulinemia, and cardiovascular diseases [24]. The lipid abnormalities are prevalent in diabetes mellitus because insulin resistance or deficiency affects key enzymes and pathways in lipid metabolism [24]. From this study, it is found that statin should be recommended to all diabetes patients with high cholesterol level. Treatment recommendations beyond lifestyle modification and optimisation of glycaemic control are for the use of high-intensity statin therapy (atorvastatin 40-80mg or rosuvastatin 20-40mg) in patients of all ages with overt coronary heart disease (CHD), and those aged 40-75 years with additional risk factors, and moderate-intensity statin therapy (atorvastatin 10-20mg or simvastatin 20-40mg) for patients aged over 40 years without any other additional risk factors. Clinical judgement should guide the use of moderate or high-intensity statin therapy in patients younger than 40 years or older than 75 years with additional risk factors [24]. Statins are the most extensively used lipid-lowering medications and are often the first choice for the treatment of diabetic dyslipidaemia. Statins primarily lower LDL levels, but they also have the secondary effects of lowering TG and increasing HDL levels. Furthermore, statins may increase the particle size of LDL to allow less circulation of smaller, dense LDL [16].

The strengths of this study include relatively a large sample size, properly planned and similar audit methods for the whole country, which makes the result of the audit representable for each state and the whole country. Besides that, the 5 years of data analysis seems to be adequate to see any significant differences in the audit results that reflect the quality of diabetes management of the state and the whole country. The few limitations of this study include the large sample size. The significance (P<0.001) could be due to the small effect of the size and large sample size. Secondly, from more than 80,000 samples extracted from the registry, more than 50,000 samples were eliminated due to it being incomplete data (no data for waist circumference, BMI, HbA1c and lipid profile). Other than that, the low awareness of the healthcare workers to take the patient´s waist circumference and BMI annually also contributed to this issue.

## Conclusion

The focus on the intervention and care need to be emphasized in diabetes management, especially for patients with high BMI, waist circumference and high total cholesterol. Statin should be started early on to ensure their glycaemic control is good as well as to reduce the risk of cardiovascular complication. Lifestyle changes, including increased physical activity and dietary modifications, are the milestones of management to reduce BMI and waist circumference and to achieve good glycaemic control. Monitoring of anthropometric measurement (waist circumference and BMI) annually and lipid profile monitoring by blood tests done in regular intervals in primary healthcare also plays an important role to detect and manage lipid abnormalities, both in diabetes and non-diabetes patients. As NDR is ongoing in the healthcare system, this study can be extended to include data from 2019 onwards and could cover other states as well.

### What is known about this topic

- Diabetes mellitus is a global public health crisis and associated with serious morbidities, including neuropathy, nephropathy, retinopathy, and obesity;
- Glycaemic control is influenced by various factors including patient-related factors, disease-related factors, treatment-related factors, healthcare provider-related factors, health facility-related factors and socioeconomic factors.

### What this study adds

- These 5 years of data analysis revealed BMI and waist circumference are found to be significantly associated with glycaemic control;
- Total cholesterol, TG and LDL showed positive correlation with glycaemic control, meanwhile HDL showed negative correlation with glycaemic control.
